# Case report: Blue rubber bleb nevus syndrome with Kasabach-Merritt phenomenon in a neonate

**DOI:** 10.3389/fped.2023.1131094

**Published:** 2023-04-19

**Authors:** Mengqi Pi, Lei Zhao, Weiyang Xu, Miao Xu, Yu Ding

**Affiliations:** Department of Interventional Hemangioma & Vascular Surgery, Children's Hospital Affiliated to Zhengzhou University, Zhengzhou, China

**Keywords:** blue rubber bleb nevus syndrome, Kasabach-Merritt phenomenon, neonate, sirolimus, glucocorticoids, treatment

## Abstract

Blue rubber bleb nevus syndrome (BRBNS) is a rare disease characterized by multifocal venous malformations that can affect any organ or tissue. Kasabach-Merritt phenomenon (KMP) is a serious and extremely rare complication of BRBNS. This report describes a neonate with BRBNS with KMP who was successfully diagnosed and treated with low-dose sirolimus and glucocorticoids. A 13-day-old female infant was born with multiple tumors on her head, neck, shoulder, back, abdomen, limbs, perineum, etc. some of which were blue. Laboratory examinations showed thrombocytopenia, anemia and coagulopathy. BRBNS with KMP was diagnosed. Oral low-dose sirolimus combined with glucocorticoids was administered. After 6 months of regular follow-up, the lesions in the child were significantly decreased, and there were no signs of KMP recurrence. The presence of KMP should be considered in patients diagnosed with BRBNS who present with thrombocytopenia, anemia and coagulopathy. Sirolimus combined with glucocorticoid therapy can be administered to save the patient's life.

## Introduction

Blue rubber bleb nevus syndrome (BRBNS) is a rare disease characterized by multiple venous malformations caused by vascular development dysplasia, with an estimated prevalence of 1 in 14,000 births (1). The lesions most frequently occur on the skin and in the gastrointestinal system, and other sites can be involved, such as the central nervous system, skeletal muscle and lung (2). The disease was first described in 1,860 by Gascoyen (2), and then Bean systematically described and named it in 1958, so it is also called Bean syndrome (3). Sirolimus is a new drug for the treatment of BRBNS, that has a good therapeutic effect and can be administered to neonates (4).

The Kasabach-Merritt phenomenon (KMP) is a rare but life-threatening coagulopathy of infancy that presents with thrombocytopenia, microangiopathic hemolytic anemia, and consumptive coagulopathy in the setting of a rapidly enlarging vascular tumor or malformation (5). The pathogenesis may be linked to platelets being captured by pathological vascular endothelial cells, which make platelets adhere and aggregate, and then activate the coagulation cascade reaction, which leads to the consumption of platelets and coagulation factors. In severe cases, disseminated intravascular coagulation (DIC) may eventually be induced. At present, glucocorticoids are still the first choice for the treatment of KMP (6).

The occurrence of BRBNS with KMP is extremely rare. Jitsuiki et al. (7) reported a similar case in a Japanese study. At present, no similar report from China has been found. Herein, we present a case of BRBNS with KMP in a 13-day-old female infant who was treated with oral low-dose sirolimus and glucocorticoids. After 6 months of regular follow-up, the lesions in the child were significantly reduced, and there were no signs of KMP recurrence.

## Case presentation

### Patient characteristics and clinical manifestations

A 13-day-old female infant was born with multiple tumors on her head, neck, shoulder, back, abdomen, limbs, perineum, etc. some of which were blue, and the size and number of tumors increased over time. The child was born naturally at full-term (G3P3) with a birth weight of 3.1 kg and no family history, and she was breastfed postnatally.

### Physical examination

**Figure 1:**
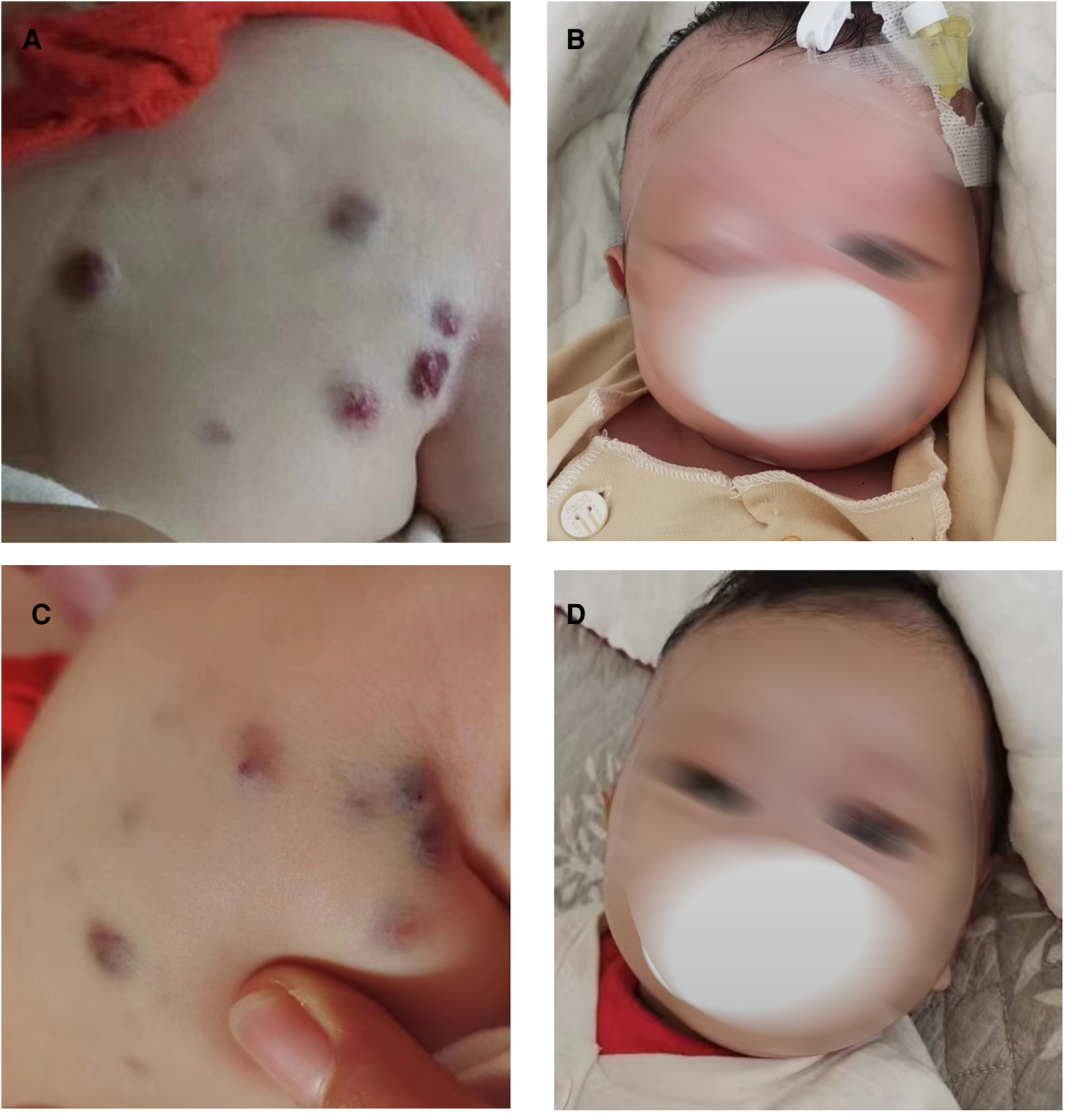
Blue rubber bleb on the back and eyes before and after low-dose sirolimus combined with glucocorticoids treatment. Blue rubber bleb on the back before treatment (**A**) and after treatment. (**C**) Blue rubber bleb on the eyes before treatment (**B**) and after treatment (**D**).

The following patient characteristics were recorded: temperature: 36.7°C, pulse: 132 times/min, respiration: 30 times/min, blood pressure: 72/64 mmHg, height: 52 cm, weight: 3.4 kg. The child appeared anemic. There were multiple tumors of different sizes on the head and neck, jaw, eyes, tongue, back (Figure 1A), abdomen, limbs, perineum and other parts, some of which were blue, soft and elastic, and compressible by palpation. Right eyelid swelling, limited upward lift, and difficulty in opening eyes were observed (Figure 1B).

### Laboratory examinations

Laboratory examinations showed thrombocytopenia,anemia and coagulopathy (Table 1).

**Table 1:** Results of blood analysis on the 13th day of life of the neonate (when the child was admitted to hospital).

Variable	Level	Unit	Normal range
Plt	96	10^9^/L	100–300
Hb	12	g/dl	18–19
Fib	0.53	g/L	2–4
PT	15.30	S	10–14
APTT	63.60	S	26–40
D-dimer	19.16	ug/ml	0–0.5
FDP	52.18	mg/L	0–5

Plt, platelets; Hb, hemoglobin; Fib, fibrinogen concentration; PT, prothrombin time; APTT, activated partial thrombin time; FDP, fibrin degradation products.

Three days after birth, the tumor of the right lateral thigh was resected and biopsied at an external hospital. The pathological report was consistent with BRBNS, and the immunohistochemical results were Glut-1 (−), CD34 (+), D2-40 (partial +), m-TOR (+), SMA (+), S100 (scattered +), and ERG (+).

The results of gene detection and analysis for genetic disease showed that no specific pathogenic gene mutation related to the subject's clinical phenotype was detected. The detection method adopts high-throughput sequencing detection technology. DNA extracted from peripheral blood was segmented and library prepared. The gene exons and adjacent regions were captured by probes, and then sequenced by high-throughput sequencing platform. The detected mutation sites were annotated by molecular biology, combined with pathogenic mutation database, normal human genome database, clinical characteristics and genetic data analysis algorithm, and finally the mutation with clinical reference significance was screened out.

### Imaging examinations

**Figure 2:**
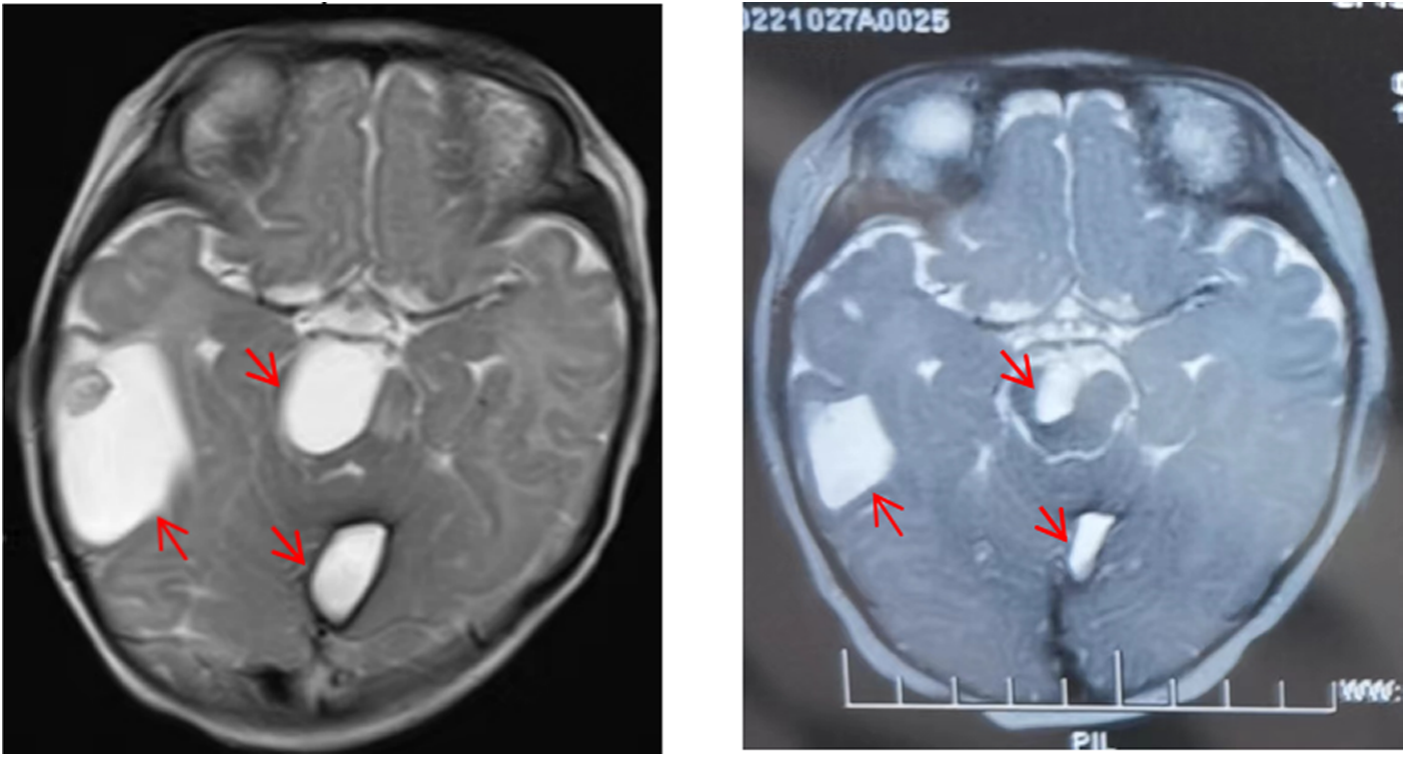
Comparison of lesions on head magnetic resonance imaging (MRI) before and after treatment. Lesions on head MRI before treatment (**A**) and after treatment (**B**).

Magnetic Resonance Imaging (MRI): examination revealed multiple systemic abnormal signals in skin (maxillofacial region, shoulder, abdomen, etc.), brain (cerebral hemisphere, thalamus, infratentorial of cerebellum, etc.) (Figure 2A), viscera (liver, lung, kidney, etc.), muscles (psoas major, iliopsoas muscle, gluteal muscle, etc.), and bone (hip, jaw, cone, etc.), which conformed to the imaging manifestations of BRBNS. Some of lesions were accompanied by hemorrhage.

### Diagnosis and treatment

**Figure 3:**
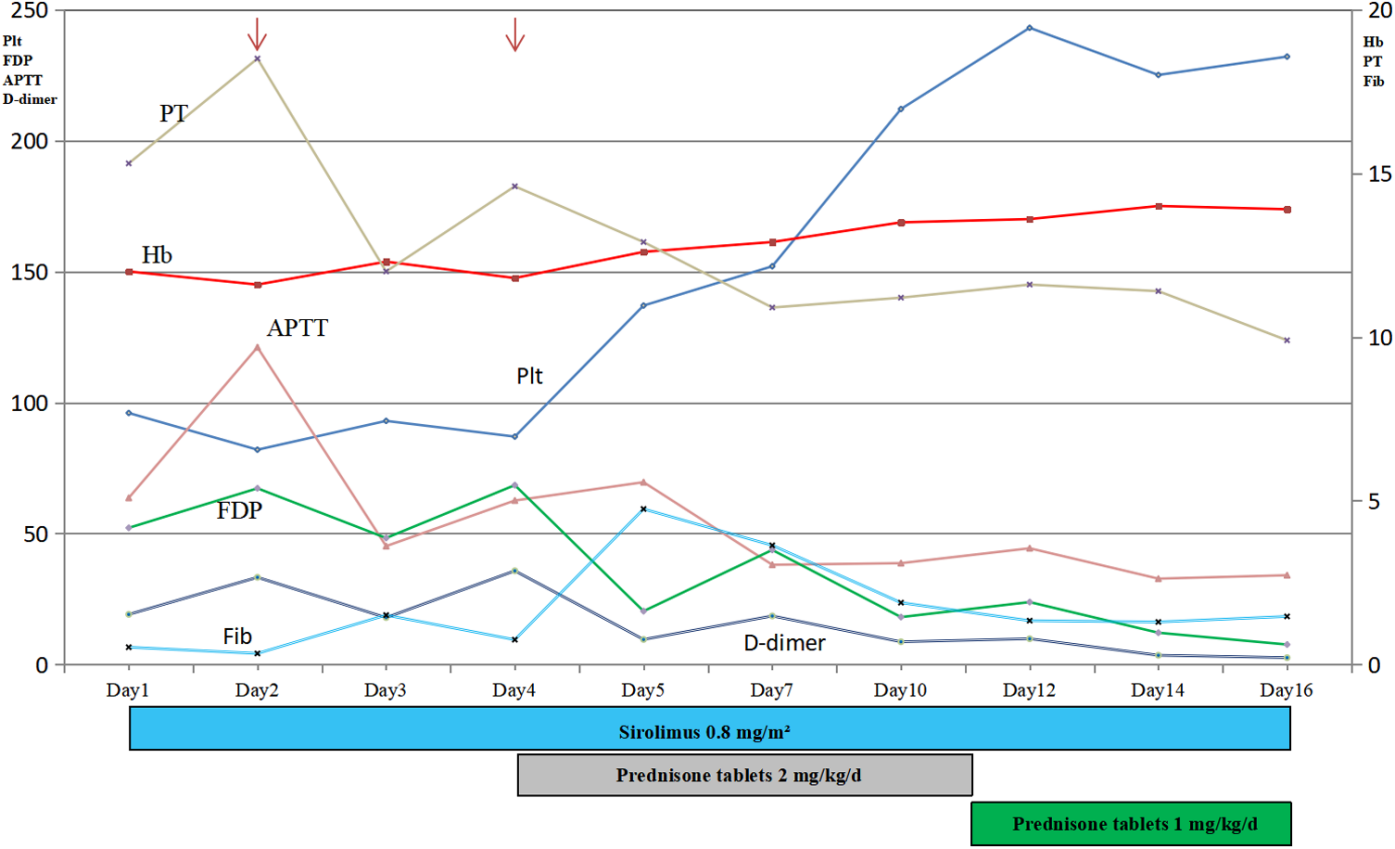
Time course of the main results of laboratory examinations and treatment. Thrombocytopenia, anemia and coagulopathy improved with treatment (arrow, transfusion). Plt, Platelets; Hb, Hemoglobin; Fib, fibrinogen concentration; PT, prothrombin time; APTT, activated partial thrombin time; FDP, fibrin degradation products.

**Figure 4:**
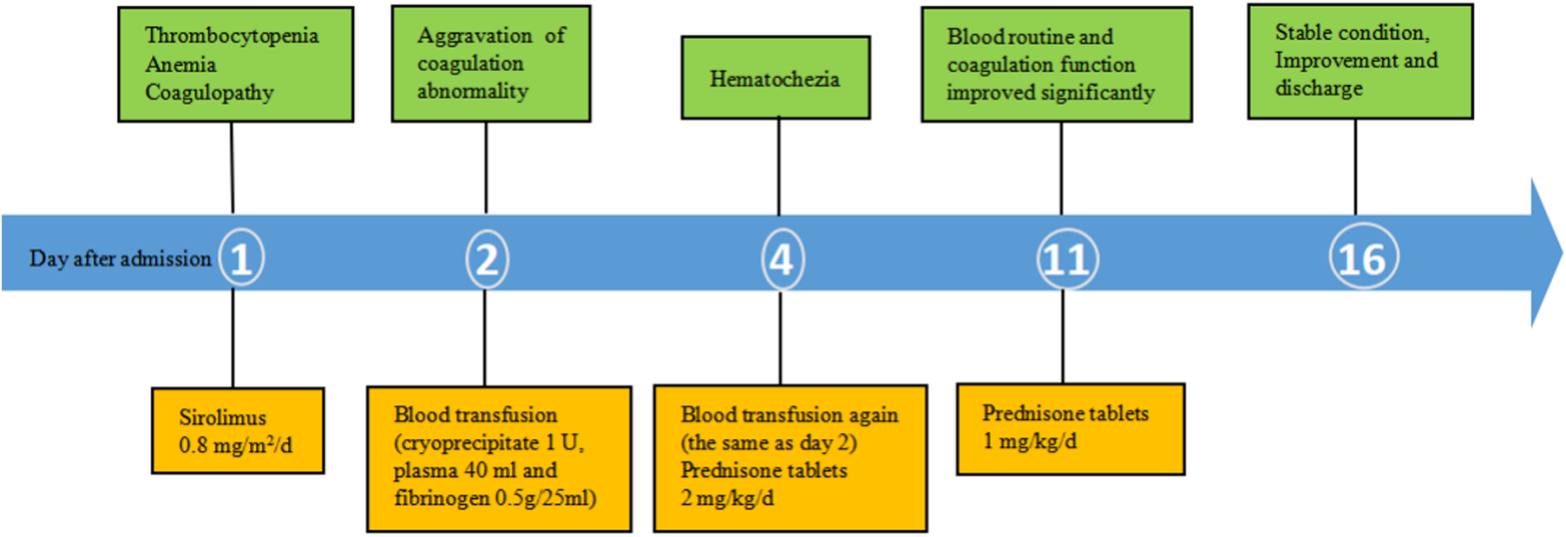
Timeline.

Combined with the above clinical manifestations and examination results, the child was diagnosed with BRBNS with KMP. She was given oral low-dose sirolimus (0.8 mg/m^2^/day) on day 1 and was transfused with cryoprecipitate 1 U, plasma 40 ml and fibrinogen 0.5 g/25 ml on day 2 due to obvious abnormalities in coagulation function (Figure 3). The child developed gastrointestinal bleeding on day 4. She was given a blood transfusion again (the same as day 2) and oral prednisone tablets at 2 mg/kg/day. On day 11, the dose of prednisone tablets was adjusted to 1 mg/kg/day, which was slowly reduced to drug withdrawal after two months (Figure 4). Coagulation function, liver and kidney function, and blood cell counts were reexamined every two weeks after discharge. MRI was performed, and the blood concentration of sirolimus was monitored every month. The blood concentration was maintained at 5–15 ng/ml. During the follow-up of 6 months, the patient did not receive a blood transfusion again, and there were no signs of KMP recurrence. The cutaneous damage was less severe than before (Figure 1C). The swelling of the right eyelid disappeared without significant limitation in lifting (Figure 1D). MRI showed that the lesions were significantly smaller than before (Figure 2B), and no significant adverse drug reactions were observed.

## Discussion

BRBNS is a rare multifocal venous malformation, and the characteristic lesions are most frequently found on the skin, in subcutaneous tissue and in the gastrointestinal tract (8). The related pathological changes are mainly vascular malformation and the formation of small lesions. Furthermore, the cutaneous lesions can be divided into 3 types: type I lesions are large and disfiguring venous malformations that progressively increase in size and can obstruct vital tissues; type II lesions, the most common BRBNS lesions, with a cyanotic appearance, are soft, elevated and easily compressible; and type III lesions are irregular blue‒black macules or papules with pigmented nevus that rarely pale under pressure (9).

Jin et al. (10) analyzed the clinical data of 120 cases reported at home and abroad, reporting that 93% (112/120) of the patients had cutaneous vascular malformations and that 76% (91/120) of the patients had gastrointestinal vascular malformations. The central nervous system (16.13%), liver (13.11%) and skeletal muscle (11.9%) were also involved. In our case, the lesions simultaneously affected multiple systems of the skin, viscera, brain, bone, muscle, and so on. BRBNS generally occurs in infancy, there is no sex difference, with a male to female ratio of approximately 1:1, and most cases are sporadic. BRBNS has been reported to be due to autosomal dominant transmission, which may be related to the mutation of genes on chromosome 9p and TEK (gene encoding TIE2) (11, 12). In our case, the child had no family history, and no pathogenic or suspected pathogenic gene mutation that could explain the clinical symptoms of the child was found through gene detection. Thus, the case involving this child may be a sporadic case.

KMP is a clinical manifestation associated with thrombocytopenia, microvascular hemolytic anemia and consumptive coagulation dysfunction, and in severe cases, it leads to disseminated intravascular coagulation (DIC) with high mortality. The possible pathogenesis involves the capture of platelets by abnormal vascular endothelial cells, and activated platelets consume coagulation factors for the second time (13). In our case, the child presented with thrombocytopenia, anemia and coagulopathy; therefore, the combination of KMP was considered. Activated platelets initiate the coagulation cascade reaction, which aggravates the consumption of platelets and coagulation factors, and ultimately leads to microvascular hemolytic anemia, coagulopathy, increased levels of D-dimer and FDP, prolonged bleeding time and coagulation time. The case of BRBNS with KMP is extremely rare and serious. Jitsuiki et al. (7) reported a similar case in a Japanese study. At present, no similar report from China has been identified.

Sirolimus is a mammalian target of rapamycin (m-TOR) inhibitor (14). m-TOR is a protein complex that contributes to several cellular processes, such as cell survival, cell growth and angiogenesis. Sirolimus is a novel and promising drug for treating BRBNS. Initially, it was approved for kidney transplantation as an angiogenesis inhibitor by the FDA in the United States. The recommended valley concentration (the lowest value of the drug after it reaches a steady state in the body) was 15–20 ng/ml. Currently, cases of BRBNS treated with sirolimus worldwide are very rare, and the dosage and duration of treatment for sirolimus are still uncertain.

However, Salloum et al. (15) reviewed 4 cases of BRBNS in children aged 2–16 years. They were all treated with oral sirolimus, 0.8 mg/m^2^, twice a day, and the blood drug concentration was controlled at 10–13 ng/ml. After 18 months of treatment, the cutaneous lesions were significantly decreased, hematochezia disappeared, and the hemoglobin level was obviously increased. Blood transfusion treatments were eliminated in all the children. Gildener-Leapman et al. (16) reported a 17-month-old child who took sirolimus 0.6 mg/day orally. The cutaneous lesions were controlled, black stool disappeared after treatment, and no obvious adverse reactions were found after 24 months of follow-up. Warner et al. (17) reported an 18-year-old child who took sirolimus 4 mg/day orally. After treatment, the hemoglobin level increased from 69 g/L to the normal range, and no obvious adverse reactions were found after 9 months of follow-up.

Low-dose sirolimus also has a better therapeutic effect on BRBNS, which can significantly reduce the size of the lesions (18), and it is also used in neonates (4). Immunohistochemistry of this patient suggested m-TOR (+). Considering the younger age of the child, she was given oral low-dose sirolimus 0.8 mg/m^2^ once a day, and the blood concentration of sirolimus was maintained at 5–15 ng/ml. After 6 months of treatment, the cutaneous damage was less severe than before. The swelling of the right eyelid disappeared without significant limitation in lifting, and MRI showed that the lesions were significantly smaller than before. In addition, there were no obvious adverse drug reactions, including those of the kidney and hepatic damage, neutropenia, systemic infection, oral stomatitis and hyperlipidemia.

Glucocorticoids can inhibit fibrinolysis and thrombosis, stimulate bone marrow hematopoiesis, increase platelet release into the blood, reduce anti-platelet antibody levels, and increase platelet counts. At present, glucocorticoids are still the first choice for the treatment of KMP (6). She was given oral prednisone tablets at 2 mg/kg/day on day 4. The dose of prednisone tablets was adjusted to 1 mg/kg/day on day 11, which was slowly reduced to drug withdrawal after two months. Coagulation function, liver and kidney function, and blood cell counts were reexamined every two weeks after discharge. After 6 months of regular follow-up, the child showed no signs of hematochezia or KMP recurrence and did not receive blood transfusion treatment again.

## Conclusion

The case of BRBNS with KMP is extremely rare and is characterized by systemic multifocal venous malformations with thrombocytopenia, anemia, and coagulopathy. Low-dose sirolimus combined with glucocorticoids has a good therapeutic effect in neonatal BRBNS with KMP patients, and there are no obvious adverse reactions.

## Data Availability

The original contributions presented in the study are included in the article, further inquiries can be directed to the corresponding author.
